# Diagnosis and follow-up evaluation of central nervous system vasculitis: an evaluation of vessel-wall MRI findings

**DOI:** 10.1007/s00415-021-10683-7

**Published:** 2021-07-08

**Authors:** Maximilian Patzig, Robert Forbrig, Clemens Küpper, Ozan Eren, Tobias Saam, Lars Kellert, Thomas Liebig, Florian Schöberl

**Affiliations:** 1 Insitute of Diagnostic and Interventional Neuroradiology, Ludwig-Maximilians-University Munich, Munich, Germany; 2 Department of Neurology, Ludwig-Maximilians-University Munich, Munich, Germany; 3 Institute of Clinical Radiology, Ludwig-Maximilians-University Munich, Munich, Germany; 4 Radiological Center Rosenheim, Rosenheim, Germany

**Keywords:** Cerebral vasculitis, Stroke, Vessel wall imaging, MRI, Follow-up

## Abstract

**Objective:**

To approach the clinical value of MRI with vessel wall imaging (VWI) in patients with central nervous system vasculitis (CNSV), we analyzed patterns of VWI findings both at the time of initial presentation and during follow-up.

**Methods:**

Stenoocclusive lesions, vessel-wall contrast enhancement (VW-CE) and diffusion-restricted lesions were analyzed in patients with a diagnosis of CNSV. On available VWI follow-up, progression, regression or stability of VW-CE were evaluated and correlated with the clinical status.

**Results:**

Of the 45 patients included, 28 exhibited stenoses visible on MR angiography (MRA-positive) while 17 had no stenosis (MRA-negative). VW-CE was found in 2/17 MRA-negative and all MRA-positive patients (*p* < 0.05). 79.1% (53/67) of stenoses showed VW-CE. VW-CE was concentric in 88.3% and eccentric in 11.7% of cases. Diffusion-restricted lesions were found more frequently in relation to stenoses with VW-CE than without VW-CE (*p* < 0.05). 48 VW-CE lesions in 23 patients were followed over a median time of 239.5 days. 13 VW-CE lesions (27.1%) resolved completely, 14 (29.2%) showed partial regression, 17 (35.4%) remained stable and 4 (8.3%) progressed. 22/23 patients received immunosuppressive therapy for the duration of follow-up. Patients with stable or progressive VW-CE were more likely (*p* < 0.05) to have a relapse (14/30 cases) than patients with partial or complete regression of VW-CE (5/25 cases).

**Conclusion:**

Concentric VW-CE is a common finding in medium/large-sized vessel CNSV. VW-CE might represent active inflammation in certain situations. However, follow-up VWI findings proved ambiguous as persisting VW-CE despite immunosuppressive therapy and clinical remission was a frequent finding.

**Supplementary Information:**

The online version contains supplementary material available at 10.1007/s00415-021-10683-7.

## Introduction

Central nervous system (CNS) vasculitis is a rare disease characterized by different etiologies, heterogeneous findings and a lack of definite diagnostic markers. Thus it poses great challenges regarding both diagnosis and treatment [1–6]. Since therapy usually consists of long-term immunosuppression with potential major adverse effects [5, 6], it is essential to establish a definite diagnosis and to evaluate the treatment response carefully. Along with clinical and laboratory findings, imaging is crucial in the work-up of CNS vasculitis [7]. However, findings of both digital subtraction angiography (DSA) and conventional magnetic resonance imaging (MRI) including magnetic resonance angiography (MRA) are unspecific regarding CNS vasculitis [8–10]. While evidence of systemic vasculitis or CNS infection can help establish the diagnosis of CNS vasculitis in some cases, brain biopsy is considered mandatory to prove primary angiitis of the central nervous system (PACNS) [5, 7, 11, 12]. Yet even biopsy has a limited sensitivity with a relevant rate of false negative results, particularly when only medium- and/or large-sized vessels are affected [13–17].

Therefore, advances regarding diagnostic tests for CNS vasculitis are required. MRI with dedicated vessel wall imaging has been proposed in this respect [18–20]. Vessel wall imaging uses different techniques to suppress the signal of intraluminal blood (“black blood imaging”), thus allowing evaluation of the vessel wall and possibly the detection of inflammatory changes within the vessel wall [19, 21]. Vessel wall contrast enhancement has been reported as a potential sign of CNS vasculitis as early as 2008 [22]. Vessel wall contrast enhancement in patients with CNS vasculitis is presumed to be caused by hyperpermeability of the endothelium and/or by neovascularization, resulting in leakage of contrast into the arterial wall either from the lumen of the main vessel or from vasa vasorum [19]. Vessel wall imaging is now used in suspected CNS vasculitis in many neurovascular centers [19]. However, there is still an eminent lack of original research on this subject, which is certainly due to the rarity of the disease. To date, there are only a few case reports and series evaluating vessel wall imaging in CNS vasculitis, with 29 patients being the largest reported CNS vasculitis group to our knowledge [23]. Even less data is available concerning follow-up MRI with vessel wall imaging in CNS vasculitis patients. According to our literature research, the largest study groups in which follow-up vessel wall imaging results were specifically reported comprise only six patients [24, 25]. Thus the role of vessel wall imaging both regarding the diagnosis of CNS vasculitis and monitoring disease activity, particularly in response to immunosuppression, remains largely unproven.

It is for these reasons that we retrospectively evaluated clinical and radiological data of patients with CNS vasculitis treated at our institution, aiming to contribute data on the pattern of vessel wall imaging findings both at the time of initial presentation and at follow-up.

## Methods

### Patients

We searched the electronic medical records of the Department of Neurology of our institution from September 2008 to December 2019 for adult patients (≥ 18 years) with suspected CNS vasculitis. The time span was chosen because dedicated vessel wall MRI has been used at our institution since September 2008.

**Fig. 1:**
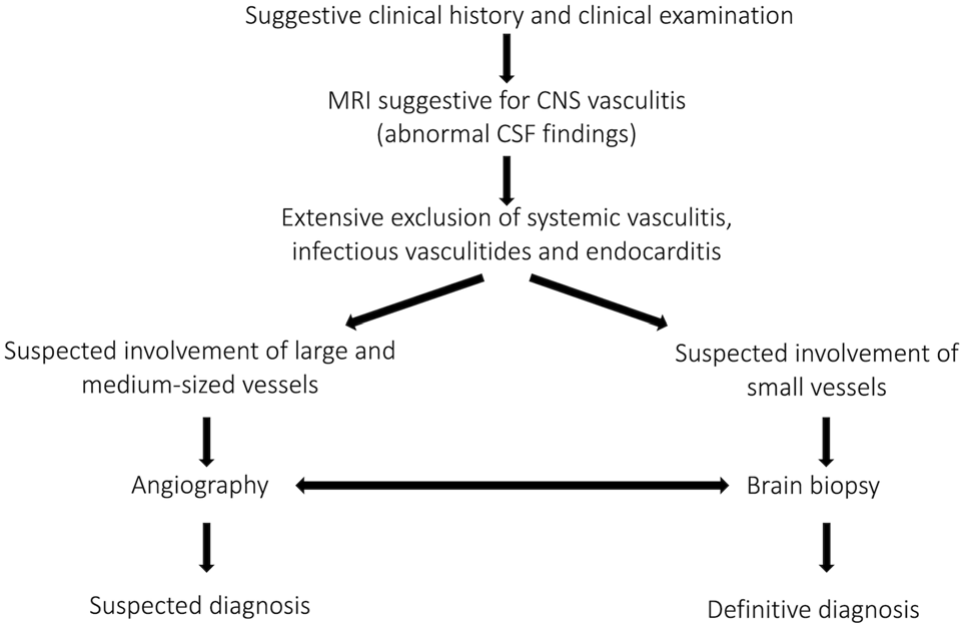
Flow chart on the diagnostic work-up for PACNS (adapted from Berlit and Kraemer [3]; Birnbaum and Hellmann [7])

In a second step, the diagnoses of CNS vasculitis were reviewed. For the purpose of this study, CNS vasculitis was defined as an inflammatory vasculopathy of cerebral arteries, either restricted to the CNS or as part of a systemic disease. The latter included CNS-manifestations of systemic autoimmune mediated vasculitis (for a systematic classification see Table 1) and vasculitic changes associated with viral meningoencephalitis. In contrast, vasculitides as a manifestation of bacterial and/or fungal meningoencephalitis were excluded, as imaging, treatment, and particularly the time course, prognosis and thus follow-up regimes for these conditions differ significantly from the included conditions. The clinical, laboratory, imaging and neuropathological data of each patient were evaluated. Relevant clinical and laboratory findings included the clinical presentation, patient age, the presence of CNS inflammation evidenced by cerebrospinal fluid exams, serologic results including parameters for systemic collagenosis and vasculitis, other evidence of systemic disease and cardiovascular risk profile. Available imaging exams [MRI, MRA, DSA, computed tomography (CT), positron-emission tomography—computed tomography (PET-CT)] were assessed for the presence, distribution and age of ischemic or hemorrhagic brain lesions, cerebral parenchymal or meningeal contrast enhancement, irregularities, stenoses or occlusions of intracranial arteries and signs of systemic vasculitis. The available neuropathological reports on brain and/or meningeal biopsies were reviewed. Patients were included for further analysis if a diagnosis of definite or probable CNS vasculitis could thus be established. Regarding PACNS, diagnoses were made according to the work-flow suggested by Berlit and Krämer [3]. This work-flow was developed with regard to the diagnostic criteria of PACNS developed by Birnbaum and Hellmann in their 2009 revision [7] of the Calabrese and Mallek criteria [11] (for details see Fig. 1). Patients were categorized according to the affected vessel size as proposed by Küker [26]: DSA-negative patients were classified as having small-vessel CNS vasculitis while patients with stenoses visualized on DSA and/or MRI were classified as having large- and/or medium-sized vessel CNS vasculitis. The large/medium vessel CNS vasculitis group was further subdivided in patients with pathologic findings (luminal irregularities, stenoses, occlusions) visible on MR angiography (“MRA-positive”) and patients with luminal abnormalities only depicted by DSA (“MRA-negative”).

**Table 1:** Classification of systemic vasculitides (adapted from the guidelines for CNS vasculitis of the German Neurological Society [27] and the Chapel Hill Consensus Conference Nomenclature [28])

Affected vessel type	Type of vasculitis
Large vessels	Giant cell arteritis (GCA) Takayasu arteriitis (TA)
Medium vessels	Polyarteritis nodosa (PAN) Kawasaki disease (KD)
Small vessels ANCA-associated	Granulomatosis with polyangiitis (GPA) Microscopic polyangiitis (MPA) Eosinophilic granulomatosis with polyangiitis (EGPA)
Small vessels associated with immune complexes	Cryoglobulin associated vasculitis (CV) Behcet’s disease (BD) Connective tissue disease Systemic lupus erythematosus (SLE) Mixed connective tissue disease (MCTD) Sjogren syndrome (SS)

Subsequently a search of the identified patients in the local Picture Archiving and Communication System (PACS) was performed. Patients who had at least one MRI scan including dedicated vessel wall imaging were included in the study.

### MRI protocol

89% of the analyzed MRI scans were acquired on a 3 Tesla Signa Excite scanner (GE Healthcare, Milwaukee, WI, USA), 8% on a 3 Tesla Magnetom Verio scanner (Siemens Healthineers, Erlangen, Germany) and 3% on a 1.5 Tesla Magnetom Aera scanner (Siemens Healthineers, Erlangen, Germany). The 1.5 Tesla scanner was used for one patient with a cardiac pacemaker not approved for 3 Tesla.

Vessel wall imaging was performed using a fat- and blood-suppressed 2D double inversion recovery spin-echo T1-weighted sequence pre- and post contrast. The sequences were acquired in axial and coronary planes with a slice thickness of 2 mm. Depending on the scanner, the in-plane resolution was 0.5 × 0.5 mm (Signa), 0.4 × 0.4 mm (Verio) or 0.9 × 0.9 mm (Aera). Eight to 14 slices were positioned to include the most prominent stenoses as visualized by Time-of-Flight-MRA. If there were no obvious stenoses, the sequences were positioned to cover the Circle of Willis.

The imaging protocols further included diffusion-weighted imaging (DWI), fluid-attenuated inversion recovery (FLAIR), T2* or susceptibility-weighted imaging (SWI) and, in most cases, contrast-enhanced MR angiography and a 3D T1-weighted sequence pre- and post contrast.

### Image analysis

Of each patient, all MRI scans including vessel wall imaging acquired within the first two years after initial presentation to our hospital and the last available MRI scan were evaluated. The timing of the initial vessel wall imaging examinations in respect to the time of presentation to our institution as well as the start of immunosuppressive therapy was documented. Follow-up intervals were categorized as “short-term” (within three months after the first MRI with vessel wall imaging), “mid-term” (3–12 months) and “long-term” (> 12 months).

All stenoocclusive lesions depicted on vessel wall imaging were recorded by correlating vessel wall imaging with MRA sequences. The lesions were graded by visual inspection of MRA as 1 = slight narrowing and irregularity of the lumen (estimated stenosis grade < 30%); 2 = moderate stenosis (30–69%); 3 = high-grade stenosis (70–99%) or 4 = occlusion. For each depicted stenoocclusive lesion, the degree of vessel wall contrast enhancement was documented as either 0 = no enhancement, 1 = moderate enhancement or 2 = strong enhancement as defined and shown by Pfefferkorn et al. [29]. Vessel wall contrast enhancement without stenosis was also recorded. Furthermore, the morphology of contrast enhancement on the initial MRI scan was classified as either concentric or eccentric. This classification was performed as previously described by Obusez et al. [25]: Enhancement was recorded as concentric if it was uniform and involved the entire circumference of the arterial wall and as eccentric if it was nonuniform, mainly on one side of the arterial wall and not involving the entire circumference. DWI was examined and diffusion-restricted lesions signifying acute ischemic infarctions were identified. The diffusion-restricted lesions were recorded according to their location in relation to the stenoses depicted on vessel wall imaging, i.e. whether they were sited in a territory supplied by a stenotic artery. In patients with contrast-enhancing vessel wall lesions, each follow-up MRI scan with vessel wall imaging was compared to the previous exam and regression, stability or progression of each contrast-enhancing vessel wall lesion was reported. Newly occurred contrast-enhancing vessel wall lesions were recorded as progression. Lesions which continued to display complete resolution of vessel wall contrast enhancement were categorized together with regressive contrast-enhancing vessel wall lesions. Diffusion-restricted lesions were also recorded on follow-up.

Vessel wall imaging on initial presentation and follow-up was analyzed by two neuroradiologists separately and blinded to other imaging and clinical findings. Discrepant reading results were resolved in consensus.

### Clinical analysis

The medical records of each patient were evaluated and clinical parameters were documented for each patient at the time of each MRI examination. At baseline, the onset of symptoms (i.e. acute versus subacute), the range of neurological symptoms (i.e. headache, focal neurological deficits, cognitive/behavioral changes, newly occurring symptomatic epilepsy/epileptic seizures) and the National Institute of Health Stroke Scale (NIHSS) Score were documented. At each follow-up, the range of neurological symptoms and the NIHSS were again documented (for details see Table 2, Suppl. 1).

**Table 2:** Characteristics of the study population

*n* = 45, f/m 1.5:1, 58 y [19–75] (median [range])		
Medium/large-vessel vasculitis	66.7% (*n* = 30) f/m 1.3:1	55 y [19–75]
Etiology/diagnosis		
PACNS	63.3% (n = 19)	52 y [19–75]
Systemic autoimmune vasculitis with CNS involvement	26.7% (*n* = 8)	60.5 y [38–74]
Giant cell arteriitis	16.7% (*n* = 5)	66 y [59–74]
Systemic lupus	3.3% (*n* = 1)	38 y
Unclassified systemic vasculitis	6.6% (*n* = 2)	41, 52 y
(Para)infectious CNS vasculitis	10.0% (*n* = 3)	57 y [53–68]
HIV/Lues	3.3% (*n* = 1)	68 y
HSV-1	3.3% (*n* = 1)	57 y
HHV-6	3.3% (*n* = 1)	53 y
Type of onset		
Acute (stroke-like)	66.7% (*n* = 20)	
Subacute (days to weeks)	33.3% (*n* = 10)	
Chronic progressive (months)	0.0% (*n* = 0)	
Symptoms at onset		
Headache	43.3% (*n* = 13)	
Neuropsychiatric complaints	16.7% (*n* = 5)	
Epilepsy/seizures	0.0% (*n* = 0)	
(Multi)focal deficits	66.7% (*n* = 20) NIHSS 2 [0–12]	
Relapses (of *n* = 21 patients with 48 follow-up evaluations)		
Overall number of relapses	33.3% (16/48)	
Clinical worsening and new DWI lesions	14.5% (7/48)	
New DWI lesions only	6.3%(3/48)	
Clinical worsening only	12.5% (6/48)	
Number of patients with relapses	52.4% (11/21)	
Clinical worsening and new DWI lesions	33.3% (7/21)	
New DWI lesions only	4.8% (1/21)	
Clinical Worsening only	19.0% (4/21)	
Immunosuppression (of *n* = 21 patients with 48 follow-up evaluations)		
Overall	95.0% (20/21)	
Steroids	95.0% (20/21)	
Other immunotherapies	66.7% (14/21) Cyc (*n* = 8), Aza (*n* = 5), MTX (*n* = 3), RTX (*n* = 2), Toc (*n* = 2)	
Combination of steroids plus one additional immunosuppressant	71.4% (15/21)	
Combination of steroids plus > 1 additional immunosuppressant	9.5% (2/21)	
Increase of immunotherapies in relapse	100% (16/16 relapses in 11/11 patients)	

Small vessel vasculitis	33.3% (*n* = 15) f/m 2:1	62 y [24–75]
Etiology/diagnosis		
PACNS	46.7% (*n* = 7)	62 y [46–74]
ANCA-associated systemic vasculitis	20.0% (*n* = 3)	73 y [44–75]
mPA	13.3% (*n* = 2)	73, 75 y
GPA	6.7% (*n* = 1)	44 y
Systemic lupus	13.3% (*n* = 2)	24, 69 y
Other autoimmune etiology	20.0% (*n* = 3)	62 y [54–67]
CREST	6.7% (*n* = 1)	67 y
Sjögren	6.7% (*n* = 1)	54 y
CAPS	6.7% (*n* = 1)	61 y
Type of onset		
Acute (stroke-like)	26.7% (*n* = 4)	
Subacute (days to weeks)	66.7% (*n* = 10)	
Chronic progressive (months)	6.7% (*n* = 1)	
Symptoms		
Headache	53.3% (*n* = 8)	
Neuropsychiatric complaints	46.7% (*n* = 7)	
Epilepsy/seizures	6.7% (*n* = 1)	
(Multi)focal deficits	80.0% (*n* = 12) NIHSS 3 [0–7]	
Relapses (of n = 2 patients with 7 follow-up evaluations)		
Overall number of relapses	42.9﻿% (﻿3/﻿7﻿)	
Clinical worsening and new DWI lesions	14.2% (1/7)	
New DWI lesions only	0% (0/7)	
Clinical worsening only	28.6% (2/7)	
Number of patients with relapses	100% (2/2)	
Clinical worsening and new DWI lesions		
New DWI lesions only	0% (0/2)	
Clinical worsening only	50.0% (1/2)	
Immunosuppression		
Overall	100% (2/2)	
Steroids	100% (2/2)	
Other immunotherapies	100% (2/2) RTX (*n* = 1), MTX (*n* = 1), Anakinra (*n* = 1)	
Combination of steroids plus one additional immunosuppressant	50% (1/2)	
Combination of steroids plus > 1 additional immunosuppressant	50% (1/2)	
Increase of immunotherapies in relapse	100% (3/3 relapses in 2/2 patients)	

*f* female, *m* male, *y* years, *Aza* azathioprine, *CAPS* cryoporine-associated periodic (fever) syndrome, *CREST* calcinosis cutis/raynaud syndrome/esophagus involvement/sclerodermia/teleangiectasia, *DWI* diffusion-weighted imaging, *GPA* granulomatosis with polyangiitis, *HHV-6* human herpes virus type 6, *HIV* human immunodeficiency virus, *HSV* herpes simplex virus, *mPA* microscopic polyangiitis, *MTX* methotrexate, *MMF* mycophenolate-mofetil, *PACNS* primary angiitis of the central nervous system, *RTX* rituximab, *Toc* tocilizumab

Additionally, on follow-up, the clinical status was recorded as either “stable disease/remission” or “relapse”. A relapse was defined according to Salvarani et al. [30] as either a recurrence of or worsening of symptoms of CNS vasculitis and/or evidence of new diffusion-restricted lesions or an increase of ischemic lesions on MRI. A relapse usually was associated with an increase in immunotherapy (see Table 2).

Treatment of CNS vasculitis with steroids and/or other immunosuppressive agents was documented at initial presentation and at the time of each MRI examination.

### Statistical analysis

Categorical variables were analyzed using two-sided Fisher’s exact test. To evaluate the course of vessel wall imaging findings in relation to clinical findings, the three possible vessel wall imaging outcomes (progressive, stable or regressive/no vessel wall contrast enhancement) were dichotomized in two different ways and compared to the clinical status of “remission” or “relapse”, also using two-sided Fisher’s exact test. *P* values < 0.05 were considered significant. Statistical analyses were performed using SPSS Statistics version 25 (IBM, Armonk, NY, USA).

## Results

### Population

45 Patients were included in the study. 27 patients were female and 18 were male. The median age was 58 years (range 19–75 years).

**Fig. 2:**
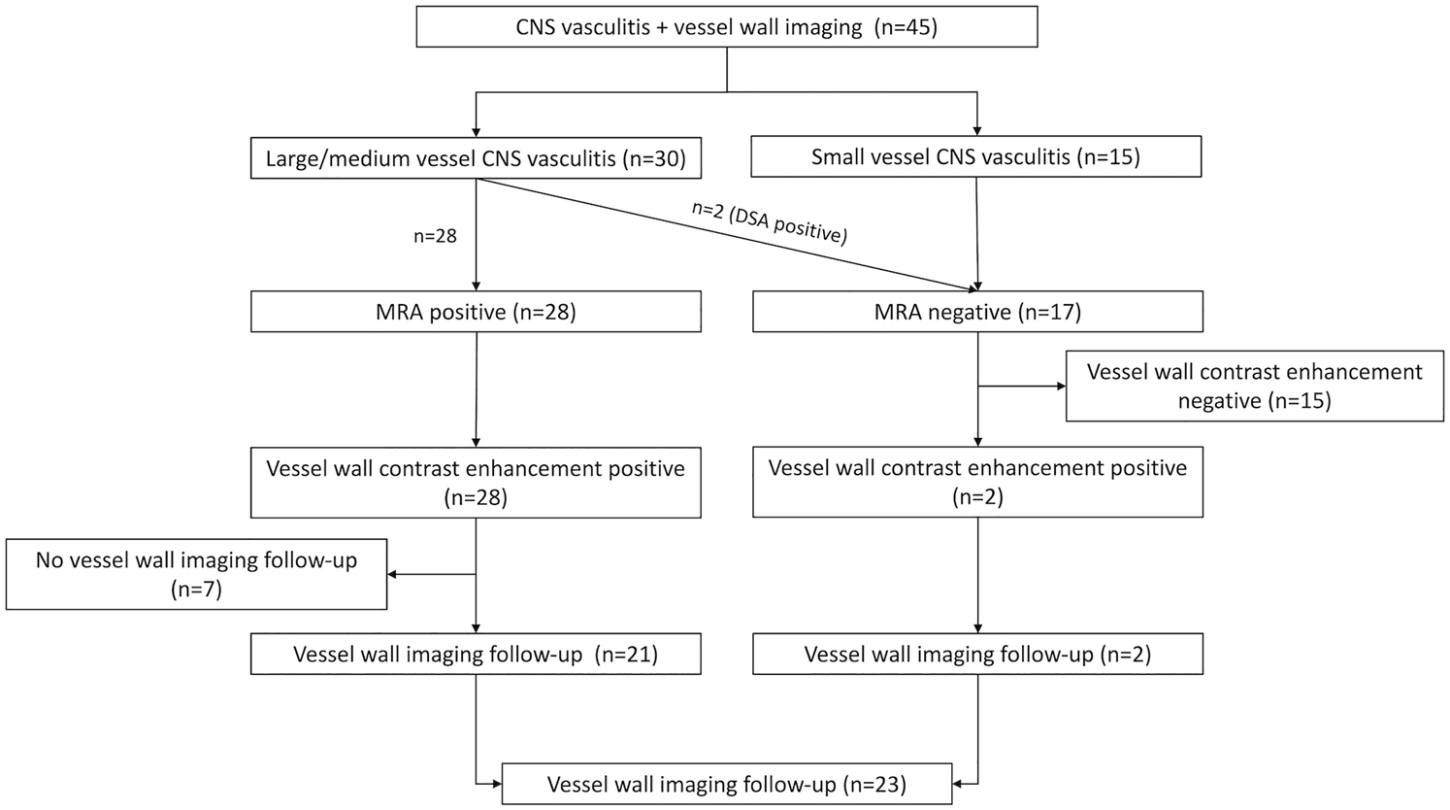
Distribution of patients to the different subgroups of the study

Initially, 15 patients were classified as small-vessel CNS vasculitis and 30 patients as large/medium vessel CNS vasculitis. In 28 of the patients with large/medium vessel CNS vasculitis, stenoses or irregularities of intracranial arteries were visualized on MR angiography (“MRA-positive”) while two patients showed abnormalities of medium-sized arteries on DSA only (“MRA-negative”). Consequently, 17 patients were initially categorized as MRA-negative and 28 as MRA-positive. Figure 2 shows the distribution of patients in the different study groups. Table 2 summarizes epidemiological and clinical data, diagnoses and treatment of the patient group.

Forty of 45 patients received their first MRI scan including vessel wall imaging within three months of the initial presentation to our institution. The other five patients were initially examined within four to eight months. Forty-four of the 45 patients were not under immunosuppressive therapy at the time of the first presentation to our hospital. All 23 patients with follow-up evaluations (see below) had their initial vessel wall imaging within four weeks of the initial presentation and none of these patients had received immunosuppression at initial presentation.

### Initial presentation

#### MRA-negative patients

15 of the 17 patients (88.2%) did not show any vessel wall contrast enhancement. Two patients (11.8%) presented with vessel wall contrast enhancement of large arteries without stenosis.

#### MRA-positive patients

28 patients harbored 67 stenoocclusive lesions examined by vessel wall imaging. The degree of stenosis was graded as “1” in eight cases (11.9%), “2” in 26 cases (38.8%), “3” in 25 cases (37.3%) and “4” in seven cases (10.4%).

53 of the stenoocclusive lesions (79.1%) showed vessel wall contrast enhancement. Vessel wall contrast enhancement without stenosis was found in seven vessel segments in five patients. Any vessel wall contrast enhancement was seen in all patients (100%). Vessel wall contrast enhancement was graded as strong in 51.7% (31/60) and as moderate in 48.3% (29/60) of cases. Vessel wall contrast enhancement was further classified as concentric in 88.3% (53/60) and as eccentric in 11.7% (7/60) of cases.

#### Group comparison

The presence of any vessel wall contrast enhancement was significantly more frequent in MRA positive vs. MRA-negative patients (*p* < 0.0001) and in large/medium-vessel CNS vasculitis vs. small-vessel CNS vasculitis (*p* < 0.0001).

#### Correlation of vessel wall imaging and diffusion-weighted imaging

Associated diffusion-restricted lesions were found significantly more often (*p* = 0.048) in stenoses with vessel wall contrast enhancement (18/50, 36.0%) than in stenoses without vessel wall contrast enhancement (1/14, 7.1%). Three stenoses were excluded from this analysis because their association to existing diffusion-restricted lesions could not clearly be determined. Diffusion-restricted lesions unrelated to visible stenoses were found in 13 patients.

### Follow-up

#### Vessel wall imaging

Twenty-three patients with 48 contrast-enhancing vessel wall lesions had follow-up MRI scans including vessel wall imaging. This comprised seven contrast-enhancing vessel wall lesions that developed during follow-up. 55 MRI scans were analyzed (*n* = 1–6 per patient) and 120 assessments of contrast-enhancing vessel wall lesions were performed overall. The length of follow-up ranged from 7 to 3543 days (Median 239.5 days).

Per contrast-enhancing vessel wall lesion and MRI scan, vessel wall contrast enhancement was graded as progressive in 10/120 cases (8.3%), as stable in 52/120 cases (43.3%) and as regressive/no enhancement in 58/120 cases (48.3%).

Per patient and MRI scan, vessel wall imaging was rated as progressive in 5/55 cases (9.1%), as stable in 25/55 cases (45.5%) and as regressive/no enhancement in 25/55 cases (45.5%).

Short-term follow-up (< 3 months) was available for 16 patients harboring 34 contrast-enhancing vessel wall lesions. Per patient, vessel wall imaging was graded as progressive in one case (6.3%), as stable in seven cases (43.8%) as regressive/no enhancement in 8 cases (50.0%).

Mid-term follow-up (3–12 months) was available for 13 patients harboring 22 contrast-enhancing vessel wall lesions. Per patient, vessel wall imaging was graded as progressive in no case (0%), as stable in three cases (23.1%) and as regressive/no enhancement in ten cases (76.9%).

Long-term follow-up (> 12 months) was available for 9 patients harboring 19 contrast-enhancing vessel wall lesions. Per patient, vessel wall imaging was graded as progressive in three cases (33.3%), as stable in one case (11.1%) and as regressive/no enhancement in five cases (55.6%).

**Fig. 3:**
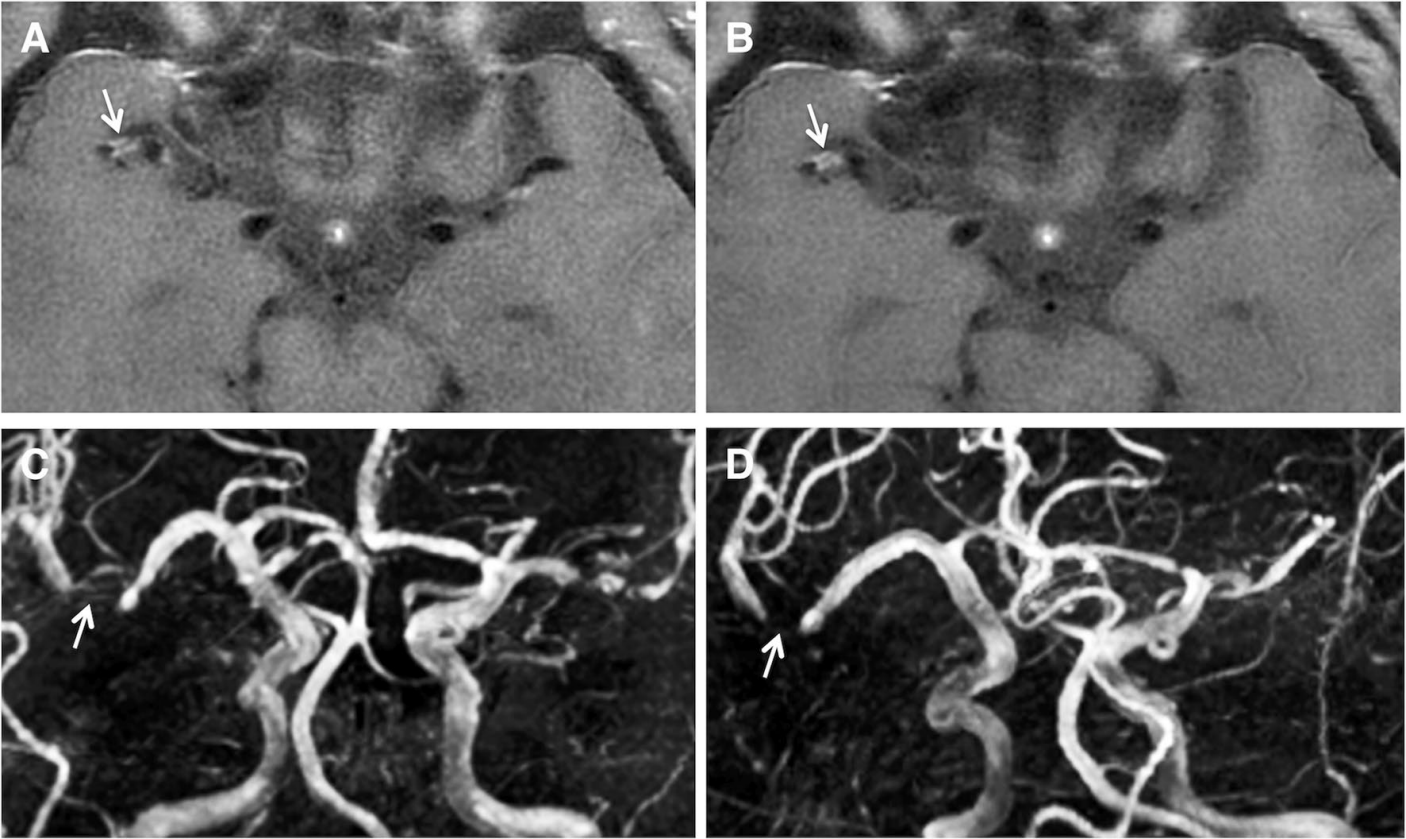
Stable vessel wall imaging findings on follow-up in a patient with PACNS. Vessel wall contrast enhancement of the right distal M1 segment is seen at initial presentation on vessel wall imaging (**A**), which remains unchanged at two-months follow-up (**B**) despite immunosuppressive therapy. Correlating TOF-MRA findings (**C**, **D**), showing unchanged high-grade stenosis of the affected segment

**Fig. 4:**
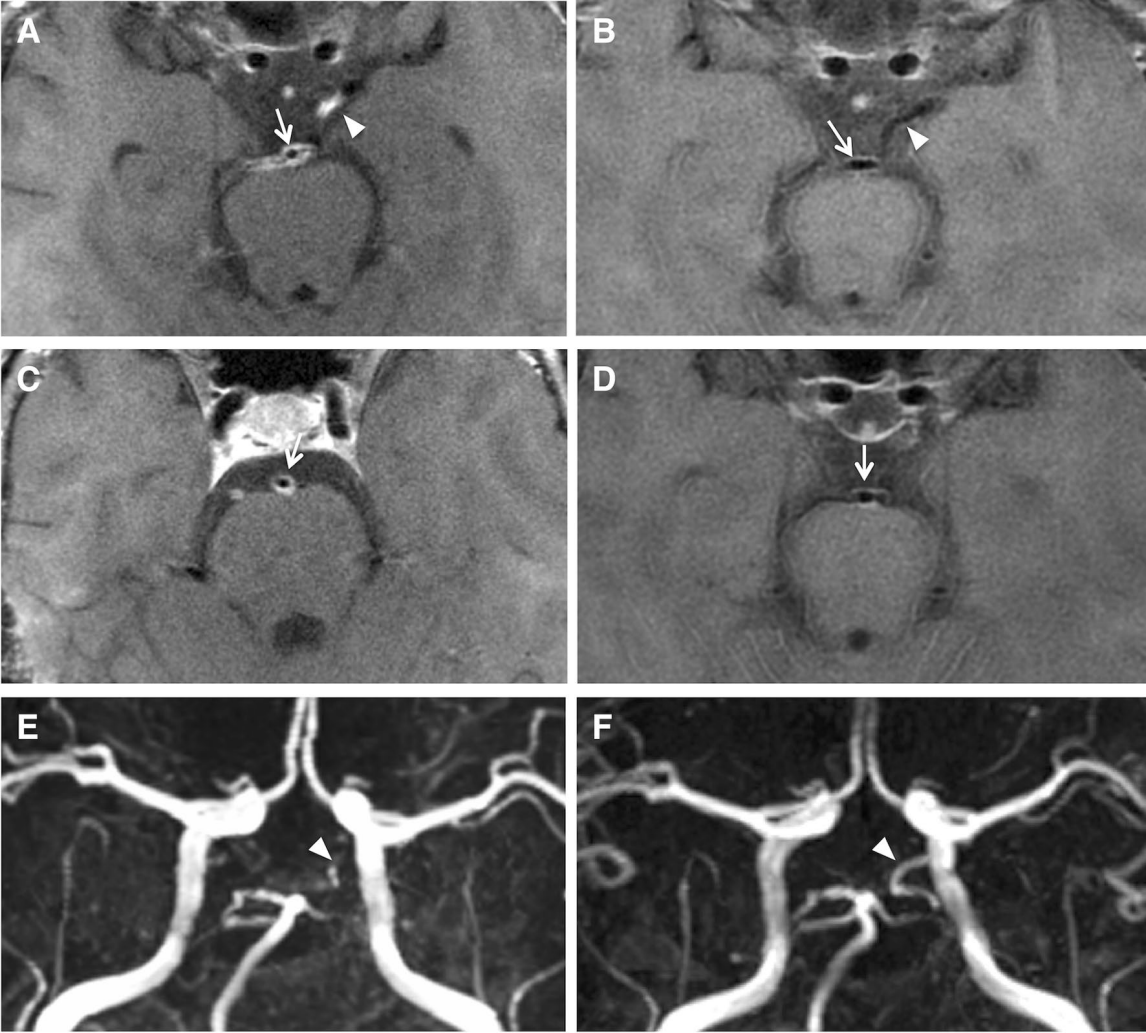
Regressive vessel wall imaging findings on follow-up in a patient with PACNS. At initial presentation (**A**, **C**), there is marked vessel wall contrast enhancement of the posterior circulation, including the basilar artery (arrow) and left posterior communicating artery (arrowhead). Follow-up vessel wall imaging after ten years (**B**, **D**) shows complete resolution of vessel wall contrast enhancement of the posterior communicating artery and regressive but still persistent vessel wall contrast enhancement of the basilar artery. Correlating TOF-MRA images (**E**, **F**) demonstrate resolution of a high-grade stenosis of the left posterior communicating artery. The findings after ten years are unchanged compared to a six months follow-up scan (not shown). The patient was under immunosuppressive therapy for the whole follow-up period

**Fig. 5:**
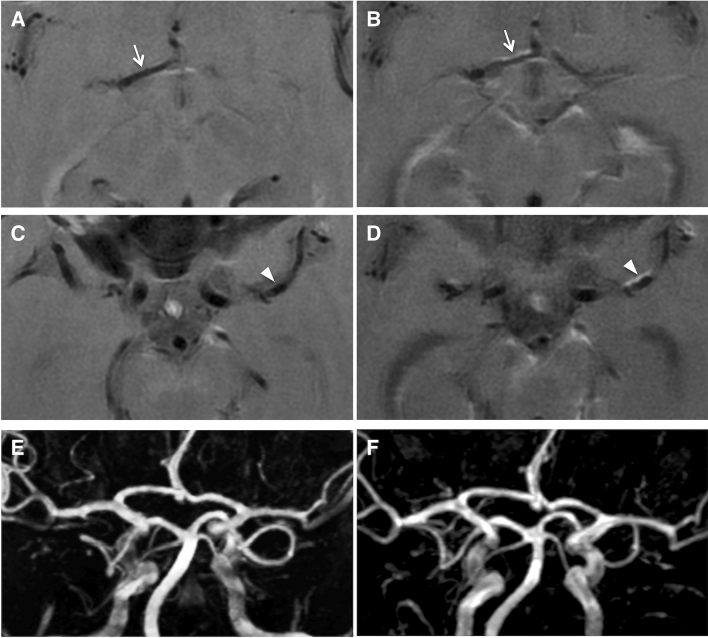
Progressive vessel wall imaging findings on follow-up in a patient with CNS vasculitis due to cryopyrin-associated periodic syndrome. Follow-up vessel wall imaging performed 34 months after the initial presentation (**B**, **D**) depicts contrast enhancement along the anterior vessel walls of the right A1 segment (arrow) and the left M1 segment (arrowhead), which was not identifiable on the initial MRI scan (**A**, **C**). Perivascular contrast enhancement surrounding the posterior cerebral arteries can be seen on both scans. Correlating TOF-MRA images (**E**, **F**) at both times do not show stenoses of the arteries of the circle of Willis (“MRA-negative”). The patient was under immunosuppressive therapy for the follow-up period

Table 3 shows the evolution of contrast-enhancing vessel wall lesions overall and at the different predefined time intervals. Figures 3, 4, 5 show examples of stable, regressive and progressive vessel wall imaging findings.

**Table 3:** Evolution of vessel-wall contrast enhancement on follow-up

Follow-up interval	Complete resolution of VW-CE	Partial regression of VW-CE	Stability of VW-CE	Progression of VW-CE
Entire Follow-up* (*N* = 48)**	13 (27.1%)	14 (29.2%)	17 (35.4%)	4 (8.3%)
Short-term (*N* = 34)**	2 (5.9%)	12 (35.3%)	19 (55.9%)	1 (2.9%)
Mid-term (*N* = 22)**	7 (31.8%)	9 (40.9%)	6 (27.3%)	0 (0%)
Long-term (*N* = 21)**	8 (38.1%)	6 (28.6%)	3 (14.3%)	4 (19.0%)

*VW-CE* vessel wall contrast enhancement

*Comparison of the initial MRI scan with the last available MRI scan of each patient

**Number of evaluated VW-CE lesions

#### Diffusion-restricted lesions

Diffusion-restricted lesions were found in nine of 23 patients and in eleven of the 55 follow-up MRI scans. On nine MRI scans, diffusion-restricted lesions were associated with a contrast-enhancing vessel wall lesion while on two scans, diffusion-restricted lesions were unrelated to contrast-enhancing vessel wall lesions (no statistically significant difference).

#### Clinical parameters and therapy

One patient with one follow-up MRI did not receive specific treatment. 22 patients with 54 MRI scans were under steroid and/or immunosuppressive treatment for the length of follow-up (see Table 2).

The status of the patients at the time of the follow-up MRI scans was assessed as “stable/in remission” on 36/55 occasions (65.5%) and as “relapse” on 19/55 occasions (34.5%).

On occasions with progressive vessel wall contrast enhancement on follow-up (*n* = 5), the clinical status was “remission” in two cases (40.0%) and “relapse” in three cases (60.0%). When vessel wall contrast enhancement was stable (*n* = 25), the clinical status was “remission” in 14 cases (56.0%) and “relapse” in eleven cases (44.0%). In cases with no vessel wall contrast enhancement or regression of vessel wall contrast enhancement findings (*n* = 25), the clinical status was “remission” in 20 cases (80.0%) and “relapse” in five cases (20.0%).

A relapse was found significantly less often (*p* = 0.0495) in cases with no or regressive vessel wall contrast enhancement (5/25 cases) compared to cases with stable or progressive vessel wall contrast enhancement (14/30 cases). There was no significant difference (*p* = 0.3273) regarding the frequency of relapses when pooling cases with no or regressive and stable vessel wall contrast enhancement (16/50 cases) and comparing them to cases with progressive vessel wall contrast enhancement findings (3/5 cases).

## Discussion

Regarding the findings at the time of initial presentation of the patients, the results of our study corroborate several common assumptions about vessel wall imaging in patients with CNS vasculitis. Any vessel wall contrast enhancement was reported by Küker et al. in 85.2% and by Thaler et al. in 60.9% of cases with large/medium vessel CNS vasculitis [22, 23]. In our study, more than three quarters of the depicted stenoses showed vessel wall contrast enhancement and any vessel wall contrast enhancement was found in each of the MRA-positive patients. Thus we confirmed that vessel wall contrast enhancement is a frequent finding in patients with large/medium vessel CNS vasculitis. However, it is important to be aware that vessel wall contrast enhancement is not exclusive to vasculitis but can also occur in various other pathologies. Based on an overview of differential diagnoses of PACNS published in a recent review article [31], we compiled a list of different subtypes of central nervous system vasculitis as well as possible differential diagnoses in which vessel wall contrast enhancement has been reported in the literature (see Table 4). From this list it becomes clear that a diagnosis of central nervous system vasculitis cannot be based simply on the presence of vessel wall contrast enhancement. Important potential mimicks of central nervous system vasculitis in which vessel wall contrast enhancement has been shown, at least to a lesser extent, include atherosclerosis, moyamoya disease and reversible cerebral vasoconstriction syndrome (RCVS) [32–36]. However, the morphologic characteristics of vessel wall contrast enhancement could be helpful in distinguishing between distinct vasculopathies. Vasculitis is usually considered to result in concentric wall-thickening and enhancement as opposed to eccentric plaque enhancement in atherosclerosis [18, 19]. Our findings support this assumption, as 90% of vessel wall contrast enhancements were classified as “concentric”. This also shows, however, that eccentric vessel wall contrast enhancement can occur in CNS vasculitis in a minority of cases, thus potentially further complicating the differentiation from atherosclerosis. Our results in this regard corroborate the findings of Obusez et al. [25] who reported eccentric wall abnormality in three of twelve CNS vasculitis cases. Further research is needed to define the role of vessel wall imaging in differentiating central nervous system vasculitis from other vasculopathies.

**Table 4:** Reports of intracranial vessel wall contrast enhancement in central nervous system vasculitis and its differential diagnoses

Disease	Reports of intracranial vessel wall contrast enhancement (examples)
Primary angiitis of the central nervous system	Own data^a^; Thaler et al. [23]; Mossa-Basha et al. [34]; Pfefferkorn et al. [29]; Küker et al. [22]
CNS vasculitis as part of a primary systemic vasculitis	
Giant cell arteritis	Own data^a^; Poillon et al. [37]; Siemonsen et al. [38]
ANCA-associated vasculitides	Own data^a^
Systemic autoimmune and rheumatic diseases	
Neurosarcoidosis	Kobayashi et al. [39]
Neuro-Behcet	Kaido et al. [40]
Systemic Lupus erythematodes	Own data^a^; Sarbu et al. [41]; Takeshita et al. [42]; Küker et al. [22]
Systemic sclerosis	Küker et al. [22]
Other autoimmune diseases	
Susac syndrome	Padrick et al. [43]; Yahyavi-Firouz-Abadi et al. [44]
Cryopyrin-associated periodic syndrome	Own data^a^
Infectious vasculopathies	
Viral infections (e.g. VZV, HSV, HIV, SARS-CoV 2)	Own data^a^; Lersy et al. [45]; Mossa-Basha et al. [34]; Cheng-Ching et al. [24], Küker et al. [22]
Basal meningitis caused by tuberculosis or fungal infections	Lu et al. [46]; Lopes et al. [47]
Bacterial infection (e.g. borreliosis, lues)	Askar et al. [48]; Bäuerle et al. [49]; Kurian et al. [50]; Lebas et al. [51]
Radiation-induced vasculopathy	Li et al. [52]
Noninflammatory vasculopathies	
RCVS	Chen et al. [32];Mossa-Basha et al. [34]; Obusez et al. [25]
Atherosclerosis	Mossa-Basha et al. [34]; Mossa-Basha et al. [33]; Skarpathiotakis et al. [35]; Lou et al. [53]
CADASIL	Goldstein et al. [54]
Moyamoya angiopathy	Wang et al. [36]; Mossa-Basha et al. [33]; Ryoo et al. [55]; Kim et al. [56]
Metabolic diseases	
Fabry disease	Kong et al. [57]
Malignant diseases	
Vascular lymphoma	Schaafsma et al. [58]

^a^Vessel wall contrast enhancement reported in this study

Thaler et al. [23] compared MRI features of large-vessel PACNS and small-vessel PACNS and found significant differences regarding the frequency of vessel wall contrast enhancement, reporting no vessel wall contrast enhancement in six patients with small-vessel PACNS. Our results are in agreement, as vessel wall contrast enhancement was also found significantly less often in small-vessel CNS vasculitis (2 of 17 cases) than in large/medium vessel CNS vasculitis. It is not a surprising result, as the spatial resolution of MRI might be too low to assess very small arteries/arterioles which cannot be evaluated on DSA. Moreover, our vessel wall imaging sequences were placed to depict large- and medium-sized arteries. However, it is an indication that vessel wall imaging usually will not show signs of large-/medium-sized vessel inflammation which is “invisible” on luminal imaging in patients with small-vessel CNS vasculitis. In our group, though, there were also two exceptions to that rule. In particular, there was one patient with cryopyrin-associated periodic syndrome (CAPS) who did not show obvious changes of large- or medium-sized arteries but presented with ischemic stroke and marked perivascular contrast enhancement of arteries of the circle of Willis (see Fig. 5). This underlines the heterogeneity of the spectrum of inflammatory vasculopathies, which makes generally applicable statements difficult.

In our group of CNS vasculitis patients, diffusion-restricted lesions were associated with vessel wall contrast enhancement of stenoocclusive lesions at initial presentation. This observation suggests that vessel wall contrast enhancement represents a condition of the vessel wall that predisposes to ischemic stroke. This in turn might indicate that vessel wall contrast enhancement initially represents active inflammation causing prothrombogenic changes in the vessel wall and/or progressive stenosis.

Vessel wall imaging has been suggested as a means to monitor disease activity and treatment response in patients with CNS vasculitis [19, 20, 59, 60]. Several case reports and figures in review articles have been published showing decreasing or completely resolving vessel wall contrast enhancement under immunosuppressive therapy [20, 21, 61, 62]. Moreover, a significant effect of corticosteroid treatment on vessel wall contrast enhancement of the superficial temporal artery in patients with giant cell arteritis has been shown [63]. However, Obusez et al. evaluated the follow-up of six CNS vasculitis patients receiving immunosuppressive treatment over a median period of 13.5 months and found resolution of enhancement in only two cases and stable enhancement in four cases [25]. Furthermore, the vessel wall imaging study group of the American Society of Neuroradiology states that, according to their experience, “there may be a discordance between intracranial VW-MR imaging findings and the clinical impression of vasculitis disease activity” [19]. Our analysis of follow-up vessel wall imaging in 23 CNS vasculitis patients largely supports this statement. Twenty-two of 23 patients received immunosuppressive therapy for the length of follow-up, which is probably the reason why progressive vessel wall imaging findings were rare. Complete resolution, regression without disappearance and stability of vessel wall contrast enhancement were relatively evenly distributed. Thus, while some patients showed quite obvious treatment response, others exhibited continued enhancement with unclear significance. Even on long-term follow-up, spanning periods of roughly one to ten years, persistence of vessel wall contrast enhancement was a frequent finding (see Fig. 3). Patients with stable or progressive vessel wall imaging findings more frequently had relapses than patients with regressive or without vessel wall contrast enhancement. In principle, this is in line with the findings at initial presentation, suggesting that vessel wall contrast enhancement might represent active inflammation. Yet there were also many cases of patients with persisting vessel wall contrast enhancement who were in remission, indicating that vessel wall contrast enhancement is not per se associated with clinical disease activity. However, vessel wall contrast enhancement in CNS vasculitis patients in clinical remission might still represent otherwise covert active inflammation of the vessel wall, as has been shown in biopsies of patients with Takayasu arteritis [64]. Alternatively, persisting vessel wall contrast enhancement could be caused by post-inflammatory mural fibrosis with or without neovascularization, as it is found in so-called “healed” giant cell arteritis [65]. As histopathologic evidence from large- or medium-sized cerebral arteries is difficult to obtain, further empiric studies will have to show whether vessel wall contrast enhancement is useful in guiding therapeutic decisions, such as possible discontinuation of long-term immunosuppressive medication. As CNS vasculitis represents an inhomogeneous group of disorders, it is also plausible that vessel wall contrast enhancement could behave differently on follow-up and under therapy in different subtypes of the disease, such as PACNS, systemic vasculitis with cerebral involvement and virus-associated vasculitides. Overall, the significance of vessel wall contrast enhancement as a biomarker of inflammatory activity on follow-up of CNS vasculitis patients remains ambiguous.

Our study is limited by its retrospective design, which for example led to varying numbers and intervals of follow-up examinations. The study group was inhomogeneous regarding the different subtypes of CNS vasculitis. Although the diagnoses of vasculitis were based on a thorough review of the cases, most of them were clinical- and imaging-based, thus the inclusion of patients with non-inflammatory vasculopathies cannot be completely ruled out. This is a problem inherent in studies including large/medium vessel PACNS patients, as the minority of published large/medium vessel PACNS cases are biopsy-proven [1, 6]. The lack of a control/comparison group precluded assertions about the value of vessel wall imaging in differentiating CNS vasculitis from other intracranial vasculopathies in our study. Another important limitation of our cohort is that five of the 45 included patients received their initial vessel wall imaging more than three months after the initial clinical presentation. Thus the initial vessel wall imaging findings cannot be interpreted with certainty to be representative of a (sub)acute state of the disease in all patients. However, in all 23 patients with follow-up MRI scans this was the case.

## Conclusions

Analyzing a comparably large group of patients, we found that concentric vessel wall contrast enhancement is common in large/medium vessel CNS vasculitis and rare in small-vessel CNS vasculitis. At initial presentation, vessel wall contrast enhancement of a stenosis was associated with an increased probability of ischemic stroke, supporting the assumption that vessel wall contrast enhancement might represent inflammatory activity. This is further substantiated by the fact that patients with stable or progressive vessel wall imaging findings on follow-up evaluations were more likely to have a relapse. However, persisting vessel wall contrast enhancement despite immunosuppressive therapy and clinical remission was also a frequent finding. Overall, follow-up vessel wall imaging findings and their clinical correlation proved ambiguous. Given the rarity of the disease, multi-center pooling of data in large patient registers will be necessary to determine whether vessel wall imaging has value in guiding treatment decisions in patients with CNS vasculitis.

## Supplementary Information

Below is the link to the electronic supplementary material.

Supplementary file1 (DOCX 25 kb)

## Data Availability

The data that support the findings of this study are available from the corresponding author on reasonable request.
