# Supplementary material for: Diagnosis and follow-up evaluation of central nervous system vasculitis: an evaluation of vessel-wall MRI findings
Source: J Neurol. 2021 Jul 8;269(2):982–96. doi: 10.1007/s00415-021-10683-7 (PMC8264821; doi:10.1007/s00415-021-10683-7)
Supplement: Supplementary file 1 — Supplementary file1 (DOCX 25 kb) [file 415_2021_10683_MOESM1_ESM.docx]

**Supplement 1. Characteristics of the individual patients of the study cohort**

| **Patient**  **Nr.** | **Age/**  **Gender** | **Diagnosis** | **Affected vessels** | **NIHSS** | **Other symptoms**** | **Initial therapy** | **Therapy during follow-up** |
| --- | --- | --- | --- | --- | --- | --- | --- |
| 1 | 70 / F | PACNS/  ABRA* | Small | 0 | Epilepsy | Ste. + MTX | No follow-up |
| 2 | 74 / M | PACNS/  ABRA* | Small | 7 | Headache, Cog./Beh., | Ste. | No follow-up |
| 3 | 62 / F | PACNS* | Small | 3 | Headache, Cog./Beh. | None | Ste. + Aza. |
| 4 | 48 / M | PACNS* | Small | 6 | Cog./Beh. | None | No follow-up |
| 5 | 58 / F | PACNS* | Small | 0 | Headache | Ste. | No follow-up |
| 6 | 46 / M | PACNS* | Small | 6 | Headache, Cog./Beh. | Ste. | No follow-up |
| 7 | 73 / M | PACNS* | Small | 0 | None | None | No follow-up |
| 8 | 73 / F | mPA | Small | 2 | None | Ste.+ Aza. | Ste. + Aza. |
| 9 | 75 / F | mPA | Small | 5 | None | Ste. + RTX | No follow-up |
| 10 | 69 / F | SLE | Small | 1 | Headache | None | No follow-up |
| 11 | 24 / F | SLE | Small | 1 | Headache | None | No follow-up |
| 12 | 54 / M | Sjögren | Small | 5 | Cog./Beh. | Ste. + RTX | No follow-up |
| 13 | 67 / F | CREST | Small | 4 | Headache, Cog./Beh. | Ste. | No follow-up |
| 14 | 61 / F | CAPS | Small | 6 | Headache | Ste. | Ste. + Ana. + MTX; Ste. |
| 15 | 44 / F | GPA | Small | 3 | Cog./Beh. | Ste. + RTX | Ste. + RTX |
| 16 | 53 / M | HHV 6 | Medium/  large | 5 | Cog./Beh. | None | Ste. + MTX; MTX |
| 17 | 52 / F | PACNS | Medium/  large | 5 | None | Ste. | No follow-up |
| 18 | 41 / M | USV | Medium/  large | 3 | None | None | No follow-up |
| 19 | 19 / F | PACNS | Medium/  large | 3 | Headache | Ste. | Ste. + Aza. |
| 20 | 22 / F | PACNS | Medium/  large | 1 | None | Ste. | Ste.; Ste. + Cyc. |
| 21 | 68 / F | PACNS | Medium/  large | 0 | None | None | None |
| 22 | 58 / F | PACNS | Medium/  large | 1 | None | None | Ste |
| 23 | 49 / M | PACNS | Medium/  large | 0 | None | MMF | No follow-up |
| 24 | 52 / M | PACNS | Medium/  large | 11 | Cog./Beh., GCS 13 | Ste. + Cyc. | No follow-up |
| 25 | 48 / M | PACNS | Medium/  large | 2 | Headache | Ste. + Cyc. | Ste. + Cyc. + RTX |
| 26 | 59 / F | PACNS | Medium/  large | 7 | Headache,  Cog./Beh. | Ste. + Cyc. | Ste. + Aza. |
| 27 | 34 / F | PACNS | Medium/  large | 2 | Headache | Ste. + Cyc. | Ste. + Cyc.; Ste. + Aza. |
| 28 | 28 / M | PACNS | Medium/  large | 0 | None | Ste. | Ste. |
| 29 | 44 / F | PACNS | Medium/  large | 0 | None | Ste. | Ste. |
| 30 | 39 / M | PACNS | Medium/  large | 6 | None | Ste. | Ste. |
| 31 | 63 / M | PACNS | Medium/  large | 8 | None | Ste. + MTX | Ste. + MTX |
| 32 | 45 / F | PACNS | Medium/  large | 0 | Headache | Ste. | Ste. |
| 33 | 63 / F | PACNS | Medium/  large | 2 | Headache | Ste. | Ste. + Cyc.; Ste. + Aza.; Aza. |
| 34 | 57 / F | HSV | Medium/  large | 5 | None | Ste. (+Aciclovir) | Ste. |
| 35 | 75 / F | PACNS | Medium/  large | 0 | Headache | None | No follow-up |
| 36 | 60 / M | PACNS | Medium/  large | 1 | None | None | No follow-up |
| 37 | 67 / M | PACNS | Medium/  large | 6 | Headache, GCS12 | Ste. + Cyc. | Ste. + Cyc. |
| 38 | 62 / F | GCA | Medium/  large | 0 | Headache | Ste. | Ste. + Cyc.; Ste. + Cyc.+ Toc; Ste. + Toc. |
| 39 | 74 / M | GCA | Medium/  large | 0 | Cog./Beh. | Ste. | Ste. + Toc. |
| 40 | 71 / M | GCA | Medium/  large | 0 | Headache | Ste. | Ste. + Aza.; Ste. + MTX; Ste. + Cyc. |
| 41 | 59 / F | GCA | Medium/  large | 2 | Headache | Ste. | No follow-up |
| 42 | 66 / F | GCA | Medium/  large | 2 | Headache | Ste. | No follow-up |
| 43 | 38 / F | SLE | Medium/  large | 3 | None | Ste. | Ste. + RTX |
| 44 | 68 / M | HIV | Medium/  large | 12 | None | None | No follow-up |
| 45 | 51 / F | USV | Medium/  large | 0 | Headache | Cyc. | Cyc. |

*Biopsy-proven ; **The following symptoms were recorded: Headache, Epilepsy, cognitive and/or behavioral impairment (Cog./Beh.), impairment of conscious level (GCS, Glasgow Coma Scale)

PACNS, primary angiitis of the central nervous system; ABRA, amyloid-beta related angiitis; mPA, microscopic polyangiitis; SLE, systemic lupus erythemtatodes; CAPS, cryopyrin-associated periodic syndrome; HHV 6, human herpes virus 6; USV, unclassified systemic vasculitis; HSV, herpes simplex virus; GCA, giant cell arteritis; HIV, human immunodeficiency virus;

Ste., Steroids; MTX, Methotrexate; Aza., Azathioprine; RTX, Rituximab; MMF, Mycophenolate-mofetil; Cyc., Cyclophosphamide; Toc., Tocilizumab.
